# Performance of indirect adherence measures for daily oral pre-exposure prophylaxis for HIV among adolescent men who have sex with men and transgender women in Brazil

**DOI:** 10.1371/journal.pone.0310861

**Published:** 2024-12-31

**Authors:** Diana Zeballos, Laio Magno, Fabiane Soares, Jony Arrais Pinto Junior, Leila Amorim, Dirceu Greco, Alexandre Grangeiro, Inês Dourado, on behalf of The PrEP15-19 study group

**Affiliations:** 1 Instituto de Saúde Coletiva, Universidade Federal da Bahia, Salvador, Bahia, Brazil; 2 Departamento de Ciências da Vida, Universidade do Estado da Bahia, Salvador, Bahia, Brazil; 3 Departamento de Estatística, Universidade Federal Fluminense, Niterói, Rio de Janeiro, Brazil; 4 Instituto de Matemática e Estatística, Universidade Federal da Bahia, Salvador, Bahia, Brazil; 5 Faculdade de Medicina, Universidade Federal de Minas Gerais, Belo Horizonte, Minas Gerais, Brazil; 6 Departamento de Medicina Preventiva da Faculdade de Medicina da Universidade de São Paulo, São Paulo, São Paulo, Brazil; University of New South Wales, AUSTRALIA

## Abstract

**Background:**

Consistent monitoring of PrEP adherence with accurate measurement tools at point-of-care could greatly contribute to reaching adolescents with poor adherence. We aimed to assess the performance of indirect adherence measures to oral PrEP among adolescent men who have sex with men (AMSM) and adolescent transgender women (ATGW).

**Methods:**

PrEP15-19 is a prospective, multicenter, PrEP demonstration cohort study that includes AMSM and ATGW aged 15–19 in three Brazilian cities. A diagnostic accuracy study was conducted using tenofovir-diphosphate (TFV-DP) concentrations in dried blood spots as the reference standard, along with three index tests: medication possession ratio (MPR), pill count, and self-report. We calculated the area under the curve (AUC) for protective TFV-DP levels (≥800 fmol/punch) and sensitivity (SE) and specificity (SP) for established cutoff points.

**Results:**

We included 302 samples from 188 participants. Most of participants were AMSM (78.7%), aged 18–19 years (80.3%), and non-whites (72.9%). The AUC was 0.59 for MPR, 0.69 for pill count, and 0.75 for self-report. When combining MPR and self-report, the AUC increased to 0.77. Sensitivity was high for the cutoff points identified by the Youden index, 80% for MPR, 92% for self-report, and 97% for pill count. However, specificities were low 40%, 46%, and 38%, respectively.

**Conclusions:**

Indirect measures were able to discriminate adolescents with good adherence. However, their performance in identifying those with low adherence might be limited, suggesting that it is necessary to initiate adherence interventions when there is no evidence of perfect adherence. Combining measures can provide wider information on adherence.

## Introduction

Oral pre-exposure prophylaxis for HIV (PrEP) with tenofovir disoproxil fumarate plus emtricitabine (FTC/TDF) is a safe and effective strategy to reduce new HIV infections among adolescents and young people [1, 2]. In 2019, PrEP was available in 77 countries, with about 626,000 people who received at least the first PrEP prescription, 69% more than in 2018 [3]. Brazilian national public health system (in Portuguese- Sistema Único de Saúde- SUS) incorporated oral PrEP in 2017 for populations at higher risk of HIV infection [4]. In 2022, adolescents aged 15 to 17 years old were included in the updated Brazilian PrEP guidelines, expanding the prevention options for this population [5].

Unlike antiretroviral therapy, PrEP has not a lifetime indication and is recommended during periods of high risk of infection, when adherence to medication is key for PrEP to work [6]. Daily oral PrEP reduces the risk of HIV infection by 96% when at least four pills per week are used [7], however achieving and sustaining protective levels of PrEP has proven to be challenging among adolescents and young individuals [1, 8, 9]. A systematic review revealed that the proportion of suboptimal adherence among youth from sexual and gender minorities was 57.0%, which is higher than the overall proportion of 41.0% and the proportion among older individuals, which was 29.1% [10]. Continuous adherence monitoring could significantly help to reach populations with lower adherence rates, thus accurate adherence measures at point of care to capture this are needed.

Different measures have been used to assess adherence to PrEP, but there is no stablished “gold standard”. Each method has its strengths and limitations [11–13]. Direct measures involve quantifying the drug in blood, cells, hair, or urine and are deemed more accurate, which is why they are frequently employed as a reference standard [13]. That is the case of tenofovir-diphosphate (TDF-DP) quantification in dried blood spots (DBS) for PrEP studies. However, its high cost and implementation complexity limit its use in the routine of health services [14]. Indirect measures that evaluate adherence by asking the PrEP users or tracking pharmacy refills and drug dispensation records emerged as a point of care options for implementation during PrEP monitoring.

Self-report is widely used, easy to collect, and low-cost. However, the concern with this measure is the overestimation of adherence due to social desirability bias or recall bias [15]. Pill count is calculated based on the pills dispensed and returned. Medication possession ratio (MPR) is estimated from pharmacy records, considering the days between visits and pills dispensed. Both measures are easy to calculate and low-cost, the limitation with these measures is that we are assessing PrEP coverage and assuming that the pills were used [11]. Studies that compared indirect measures with DBS have found that MPR, pill count, and self-report can discriminate participants with and without sufficient drug levels for protection against HIV infection [14, 16, 17], however, few studies assessed the value of these measures among adolescents. One study conducted with young MSM, reported that self-report had a low discrimination capacity [18].

Assessing adherence is fundamental to timely identify individuals who require additional support for PrEP adherence. Furthermore, there is a need for feasible and affordable methods to assess adherence among adolescents and youth. Our objective was to evaluate the performance of indirect measures for daily oral PrEP adherence, such as MPR, pill count, and self-report, compared to TFV-DP levels in DBS, among adolescent men who have sex with men (AMSM) and adolescent transgender women (ATGW) participants of the PrEP1519 study in Brazil.

## Methods

### Study design, setting, and participants

We conducted a diagnostic accuracy study using data from PrEP15-19, a PrEP demonstration cohort study of AMSM and ATGW aged 15–19 at high risk for HIV infection, residing in Salvador, Belo Horizonte or Sao Paulo, three large capital cities in Brazil. Participants self-selected into one of two arms: i) the PrEP arm, which included those who enrolled in daily oral PrEP with the TDF/FTC combination; ii) the non-PrEP arm, which included those who were eligible for PrEP but chose not to use drug prophylaxis. Both groups also received other HIV combination prevention methods (i.e., counseling, condoms, lubricant, and HIV self-test). To be included in the PrEP arm, participants must had a negative HIV test and meet at least one of the following criteria: (i) having had condomless anal sex in the past six months; (ii) having had one or more episodes of STIs in the last 12 months; (iii) having used PEP in the last 12 months; (iv) have used alcohol and/or other drugs during sexual intercourse; (v) having engaged in commercial sexual relations; (vi) having suffered violence and discrimination due to their sexual orientation. Participants received oral PrEP in a combined pill with a fixed dose of emtricitabine 200 mg (FTC) and tenofovir disoproxil fumarate 300 mg (TDF), to be taken once daily. Following visits were scheduled at baseline, 30 days, 60 days, and then every 90 days thereafter, until the end of the study in February 2022. At each visit, enough PrEP bottles containing 30 pills were dispensed to cover the days until the next visit. In addition, at each follow-up visit, a dried blood spot (DBS) sample was collected and stored to measure direct PrEP adherence afterward. More details of the study have been published elsewhere [19].

For this analysis, we included participants who initiated PrEP and had at least one follow-up visit where a DBS sample was collected and stored between February 21, 2019, in São Paulo, April 2, 2019, in Salvador, May 13, 2019, in Belo Horizonte, and December 18, 2020, at all three sites.

### Adherence measures

We used a direct measure as reference standard, the quantification of TFV-DP in DBS samples, while index tests were indirect measures including medication possession ratio (MPR), pill count, and self-report adherence.

#### Medication possession ratio

MPR was calculated using pharmacy refill records and defined as the ratio between the number of pills dispensed and the number of days between visits. MPR ranges from zero to 1. However, this ratio can exceed 1 if more medication was dispensed than needed for the period. Values equal or more than 1 indicate being full covered during the period (≥100%).

#### Pill count

Participants were asked to return their medication bottles at each follow-up visit, and the number of unused pills was counted. Pill count was then calculated as the difference between the number of pills dispensed and the number of pills returned, divided by the number of days between visits. Results were reported as percentages, with higher values indicating better adherence.

#### Self-report adherence

Self-report was assessed during clinical assessments using the following question: ’During the last month, approximately how many days have you missed your PrEP pills? Even if it has been one or many days, please tell me as it will not affect your care.’ We then subtracted the number of missed days from 30 and calculated the percentage, with 100% indicating perfect adherence.

*TFV-DP concentrations in DBS*. During all follow-up visits, blood was collected and spotted onto filter paper for DBS, and then the DBS samples were stored. The quantification of TFV-DP concentrations in DBS was conducted at the University of Colorado Antiviral Pharmacology Laboratory (Aurora, CO, USA) using liquid chromatography mass spectrometry tandem mass spectrometry (LC-MS/MS) and extracted with 50% methanol solution. A TFV-DP concentration equal to or greater than 800 fmol/punch corresponded to 4 doses/week, as reported by laboratory. TFV-DP levels were dichotomized into highly protective drug levels (>800 fmol/punch) and poorly protective drug levels (<800 fmol/punch) [20, 21].

### Sample size calculation

The sample size was defined using Tilaki’s [22] approach who proposed a calculation for validation studies in the field of health. Using a pre-established AUC of 0.7 and a marginal error of 0.10, the required sample size was 108 adolescents for each group (highly protective drug levels and poorly protective drug levels), with 80% statistical power and 95% confidence level. The quantification of TFV-DP concentrations was essayed in stored DBS samples from i) all the participants who seroconverted to HIV while receiving PrEP; ii) all ATGW, given the smaller sample size compared to AMSM; and iii) a random sample of DBS from AMSM. The DBS samples from AMSM were numbered chronologically according to the visit date, generating the follow-up visit number. We first listed the DBS samples by site and follow-up visit number, and then DBS samples were randomly selected.

### Statistical analysis

A descriptive analysis was conducted by subpopulation using the chi-square test to compare characteristics of participants included and not included. DBS data were matched with data from each indirect measure collected on the same date. If the same date was not available, we used the closest data within a 45-day range. Information of participants who did not return their PrEP bottles or did not answer the self-report question were excluded as missing data for the corresponding indirect measure and time. We compared drug levels between missing data for indirect measures and complete information using Generalized Estimating Equations (GEE) with the logit link function. The compound symmetry correlation structure was adopted for GEE. The discriminatory capacity of each indirect measure and of the combination of these measures was assessed through the computation of the Area Under the Curve (AUC) using the Receiver Operating Characteristics (ROC) curve. The optimal cutoff points were estimated using the Youden index, which identified the points with the best balance of sensitivity and specificity [23]. We compared two approaches for this analysis, the first approach was conducted using only the first available measure per participant. In the second approach we used the repeated measures for the same participant, modeling through GEE [24, 25]. As the cutoff points in the second approach are based on the probabilities of adherence estimated by the GEE adjusted from the indirect measure, we concluded that cut-off points would not be easy to apply in clinical practice. Therefore, we opted for the alternative naive approach, which involved using a single measure to estimate the cutoff points. This decision was supported by the similarity of results for AUC in both approaches. Sensitivity (SE), specificity (SP), and positive (PPV) and negative (NPV) predictive values were calculated for the best cut-off points identified by Youden Index and for cutoff points equivalent to 4-day per week use (0.6 for MPR and 57.1% for pill count and self-report) and 7-day per week use (≥1.0 for MPR and 100% for pill count and self-report). STATA software version 15 (StataCorp, 2015) and R version 4.3.1 were used for the analyses.

### Ethical issues

The PrEP1519 study was conducted according to the Brazilian (Resolution CNS no. 466, Brazil, 2012) and international research ethics guidelines, and was approved by the Research Ethics Committees (REC) of the World Health Organization (Protocol ID: Fiotec-PrEP Adolescent study), Federal University of Bahia (#3,224,384), University of São Paulo (#3,082,360) and Federal University of Minas Gerais (#3,303,594). Written informed consent (WIC) was obtained from adolescents 18–19 years. For those <18 years, each site had a different protocol according to local court decisions: in Belo Horizonte the WIC had to be signed by the parents or guardians followed by the assent form (AF) signed by the adolescents; in Salvador there were two options: i) WIC signed by a parent or guardian and AF by the adolescent; or ii) only AF signed by the adolescent when the sociopsychology team judged that their family ties had been severed or that they were at risk of physical, psychological, or moral violence due to their sexual orientation; and in São Paulo only the AF signed by the adolescents was required. All participants could withdraw consent at any stage of the process or skip any questions perceived as too sensitive, too personal, or distressing.

## Results

A total of 703 participants have initiated PrEP during the study period, 1,447 specimens of DBS were collected from AMSM and 89 from ATGW. Out of these, 302 (19.6%) DBS samples were sent for the quantification of TDF-DP, 32 samples from individuals who seroconverted, 86 samples from ATGW, and 184 samples from AMSM. The distribution of AMSM DBS samples by follow-up visit number and week of follow-up are displayed in S1 Table and the distribution of DBS Samples by collection week for all the samples are displayed in S2 Table. These DBS samples were obtained from a total of 188 adolescents, which corresponded to 26.7% of all participants enrolled in PrEP. Most of the participants included in this analysis were AMSM (78.7%), non-white (72.9%), and aged 18–19 years (80.3%) (Table 1). When comparing the characteristics of those who were included versus those who were not, significant differences were found in the study site for both AMSM (p = 0.019) and ATGW (p = 0.004) subpopulations (S3 Table). Since all DBS samples from ATGW were sent for quantification, the "not included" category for TGW refers to those who either started PrEP but did not attend follow-up visits or had DBS samples that were lost.

**Table 1:** Baseline characteristics of participants included in the accuracy analysis. PrEP1519 study, February 2019 to December 2020.

Characteristics	Total
Age	
15–17 years	37 (19.68)
18–19 years	151 (80.32)
Sub-population	
Men who have sex with men	148 (78.72)
Transgender women	40 (21.28)
Skin-color	
White	51 (27.13)
Non-White	137 (72.87)
Study site	
Belo Horizonte	53 (28.19)
Salvador	64 (34.04)
São Paulo	71 (37.77)
Schooling	
Higher education	44 (23.40)
High school or less	142 (75.53)
Not Available	2 (1.06)
Condomless anal sex	
No	63 (33.51)
Yes	125 (66.49)
Partner living with HIV	
No/Don’t know	148 (78.72)
Yes	13 (6.91)
Not Available	27 (14.36)

Matched data with indirect measures were 302 for MPR, 274 for self-report, and 104 for pill count. We observed poorly protective drug levels for majority of visits where the PrEP bottle was not returned (67.68%), and when the self-reported adherence question was not answered (82.14%) (Table 2).

**Table 2:** Comparison of included and not included values due to missing data by levels of TFV-DP in DBS. PrEP1519 study, February 2019 to December 2020.

Characteristics	Total	Quantification of TFV-DP in DBS		
		>4 days/week	< 4 days/week	p value
Self-report				0.016
Included	274 (90.73)	110 (40.15)	164 (59.85)	
Not Included	28 (9.27)	5 (17.86)	23 (82.14)	
Pill-count				0.042
Included	104 (34.44)	51 (49.04)	53 (50.96)	
Not Included	198 (65.56)	64 (32.32)	134 (67.68)	

TFV-DP = tenofovir-diphosphate. DBS = dried blood spot.

**Fig 1:**
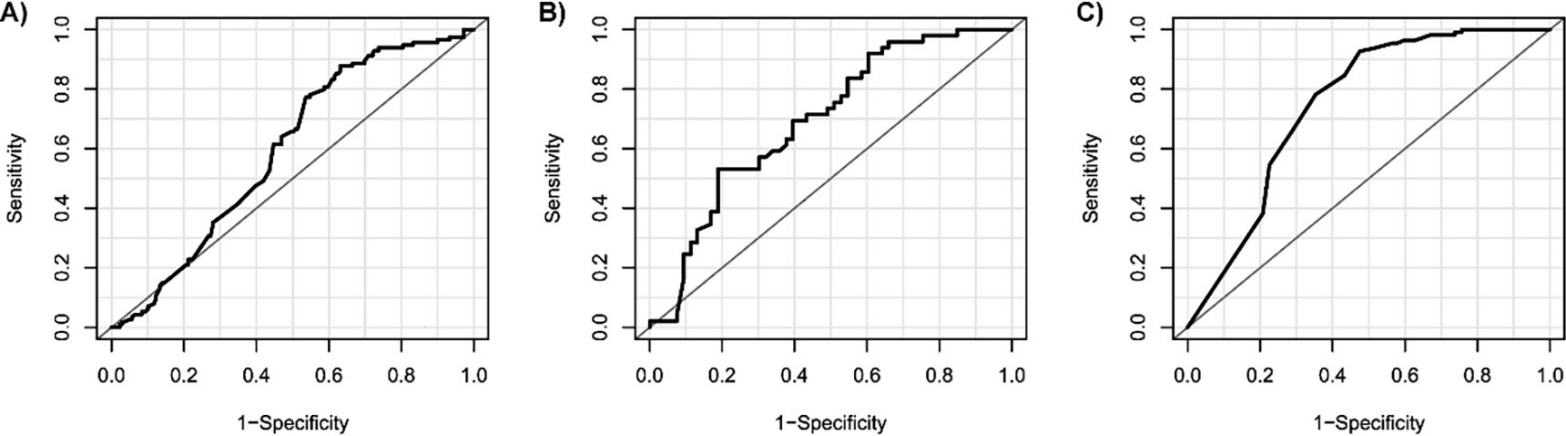
Receiver Operating Characteristics curves for indirect measures of adherence A) Medication Possession Ratio (n = 188), B) pill count (n = 68) and C) Self-report (n = 174) considering first DBS per participant. PrEP1519 study, February 2019 to December 2020.

The analysis of the ROC curve indicates that the three measures were able to discriminate those with highly protective levels of TDF-DP with AUC of 0.59 for MPR, 0.69 for pill count, and 0.75 for self-report (Fig 1 and Table 3).

**Table 3:** Accuracy of indirect adherence measures by itself and in combination, according to the first and repeated measures. PrEP1519 study, February 2019 to December 2020.

Adherence measures	n	First measure	n	Repeated measures
		AUC (95%CI)		AUC (95%CI)
MPR	188	0.55 (0.47–0.64)	302	0.59 (0.53–0.66)
Pill count	68	0.69 (0.57–0.82)	104	0.69 (0.58–0.80)
Self-report	174	0.72 (0.65–0.80)	274	0.75 (0.68–0.81)
MPR + Pill Count + Self-report	66	0.72 (0.59–0.84)	102	0.72 (0.62–0.83)
Pill Count + Self-report	66	0.71 (0.58–0.84)	102	0.73 (0.63–0.83)
MPR + Pill Count	68	0.68 (0.55–0.81)	104	0.69 (0.58–0.80)
MPR + Self-report	174	0.74 (0.67–0.82)	274	0.77 (0.70–0.83)

AUC = area under the curve; CI = Confidence interval; MPR = medication possession ratio.

Additionally, we observed that combining MPR with self-report resulted in a better discrimination capacity (AUC = 0.77). When we performed the analysis using the first measure of adherence of each participant, we found results similar to those found with repeated measures (Table 3).

The best cut-off points identify by Youden Index were 0.91 for MPR, 83.30% for self-report, and 58.70% for a pill count. Sensitivity, specificity, and predictive values for each cut-off point are displayed in Table 4.

**Table 4:** Performance of cutoff points for indirect measures of PrEP adherence. PrEP1519 study, February 2019 to December 2020.

Adherence measures	Cutoff point	Sensitivity	Specificity	PPV	NPV
**Youden index**					
MPR	0.91	0.80	0.40	0.48	0.74
Pill count	58.70	0.97	0.38	0.61	0.93
Self-report	83.30	0.92	0.46	0.55	0.89
**Equivalent to 4-days-week use or posse**					
MPR	0.60	0.95	0.17	0.44	0.83
Pill count	57.10	0.97	0.38	0.61	0.93
Self-report	57.10	0.96	0.30	0.50	0.91
**Equivalent to 7-days-week use or posse**					
MPR	1.00	0.47	0.57	0.43	0.62
Pill count	100.00	0.20	0.91	0.70	0.53
Self-report	100.00	0.37	0.80	0.57	0.64

PPV = positive predictive value. NPV = negative predictive value. MPR = medication possession ratio.

As predictive values are influenced by prevalence, we also estimated the predictive values for different scenarios of adherence prevalence (Table 5).

**Table 5:** Predictive values according with variance in prevalence of PrEP adherence.

Adherence Prevalence (%)	MPR		Pill count		Self-report	
	PPV	NPV	PPV	NPV	PPV	NPV
10	0.06	0.88	0.02	0.89	0.02	0.85
20	0.14	0.76	0.05	0.78	0.04	0.72
25	0.17	0.70	0.07	0.73	0.06	0.65
30	0.21	0.65	0.09	0.68	0.07	0.59
40	0.29	0.54	0.13	0.58	0.11	0.49
60	0.48	0.34	0.25	0.38	0.22	0.30
80	0.71	0.16	0.48	0.18	0.43	0.14

PPV = positive predictive value. NPV = negative predictive value. MPR = medication possession ratio.

## Discussion

Indirect measures for monitoring PrEP adherence among adolescents have proven effective in identifying individuals who maintained sufficient PrEP levels to prevent HIV infection at various follow-up points. Among these measures, self-report demonstrated the highest performance. Combining different adherence measures, such as self-report and MPR, provides a more comprehensive understanding of an adolescent’s adherence to PrEP. In selecting a cut-off point to determine adequate adherence using indirect measures, we found that a threshold equivalent to taking four pills per week offered high sensitivity but low specificity. This suggests that while indirect measures are effective in identifying those with good adherence, they are less capable of detecting those with poor adherence. To accurately identify adolescents with poor adherence, a cut-off point equivalent to perfect adherence would be necessary. Given their low cost and proven ability to differentiate users with high protective levels, these indirect measures can be valuable as point-of-care screening tools in PrEP programs, though it is essential to consider their limitations.

Although the literature on PrEP adherence often describes self-report as a method with poor performance that overestimates adherence, we found that self-report was the most effective measure in identifying adolescents who maintained enough PrEP levels to prevent HIV infection. In the iPrEX study among MSM and TGW, two self-report adherence collection strategies were employed, through yielding AUCs of 0.51 and 0.52 [26]. However, in the PrEP Brazil study, self-reports were able to discriminate participants with high protective levels (AUC = 0.65) [14]. The findings of a systematic review revealed no important difference between suboptimal adherence as measured by self-reports and tenofovir concentrations, suggesting that self-reports act as a convenient and affordable approximation to PrEP adherence [10]. We hypothesized that the PrEP1519 multidisciplinary team created a trustworthy friendly environment where participants felt confident in disclosing their adherence without fear of being judged. This environment was further emphasized by peer navigators who helped improve linkage to services [27]. Self-report is a simple and inexpensive method, making it feasible to be used to assess adherence. Additionally, the quality of information obtained through self-report measures could be enhanced using strategies to reduce desirability bias or facilitate recall [15].

Studies that evaluated the performance of MPR used data from specific time periods, for example, PrEP Brazil assessed participants who attended the 48th week [14], and iPrEx used data from the 24-week [26], both studies showed that MPR has a better ability to discriminate drug detection levels than self-report and pill count. In contrast, we used data from different time points, which could have impacted its performance, due to the periods of discontinuation and restarting of the PrEP use.

One disadvantage of using pill-count is compliance with returning the bottles. This behavior may prevent providers from calculating adherence [13]. We observed that participants who did not return their bottles were more likely to have drug levels below the protective threshold. In the iPrEx study, pill count performed poorly [26], although in PrEP Brazil it was able to discriminate participants with good adherence [14]. A study using pill count with unannounced visits to participants also found that it performed poorly (AUC = 0.54) and had a poor correlation with more objective measures [28].

We observed an improvement in performance with the combination of MPR and self-report. Previous studies have evaluated the additional value of combining multiple adherence measures. For example, combining self-report with more accurate measures, such as quantification of TDF in hair or plasma, which may improve the ability to identify patients with good adherence [29]. Therefore, when possible, the use of multiple adherence measures is recommended, as different measures may increase the period of adherence being evaluated.

Sensitivity for cutoff points identified by Youden index were high (SE> = 0.8), indicating that most adolescents with high protective drug levels were identified as having high adherence by indirect measures. However, those cutoff points were less specific (SP<0.5), meaning that adolescents with poorly protective drug levels had a low probability of being identified as having poor adherence with indirect measures. The positive predictive values indicated that participants identified as having high adherence had probabilities of 48%, 55%, and 61% of having high drug protective levels, assessed with MPR, self-report, and pill count, respectively. Finally, indirect measures had high negative predictive values (NPV>0.7), meaning that when adolescents were identified as having poor adherence with indirect measures, the probability of having low protective drug levels was high. Furthermore, results showed that when the prevalence of adherence increases, the ability of indirect measures to identify adolescents with poor adherence will diminish.

Our study has some limitations. First, measures were paired with those collected on different days, although we tried to avoid large gaps between measurement times. Second, the limited number of repeated measures due to our sampling process prevented us from exploring longitudinal variables more extensively, besides there is a methodological challenge related to the interpretation of estimates obtained using GEE for clinical practice. Further statistical research and developments are needed to assess accuracy and calculate cut-off points when using repeated measures. Finally, the self-report question considered the last 30 days, even if the interval between visits was longer. Therefore, our results are specific to this timeframe. Different questions, such as those considering the entire period between visits or the last 7 days, will require their own validation.

## Conclusions

Adherence monitoring is critical to improve the effectiveness of PrEP. We found that self-report, MPR and pill count perform well in identifying adolescents taking enough PrEP to achieve adequate levels of protection. However, their performance in identifying those with low adherence might be limited, suggesting that it is necessary to initiate adherence interventions when there is no evidence of perfect adherence. Further research is needed to develop or identify measures that can detect adolescents with poor adherence or those at risk of poor adherence. In the meantime, self-report and MPR remain valuable tools for monitoring real-world PrEP use and for identifying adolescents who need additional support with PrEP adherence. Furthermore, we observed that a combination of measures will add value to the use of these measures, which are easy to implement at point-of-care PrEP programs within national health systems, such as in the Brazilian SUS.

## Supporting information

S1 Table Distribution of DBS collection weeks by follow-up visit number for randomly selected AMSM. (DOCX)

S2 Table Distribution of samples per weeks among all the samples of DBS. PrEP1519 study, February 2019 to December 2020. ^a^Interval notation is used to describe categories: parentheses indicate that the number is excluded from the interval, while square brackets indicate that the number is included in the interval.
(DOCX)

S3 Table Comparison of participants’ baseline characteristics included and not included in the accuracy analysis, by subpopulation. PrEP1519 study, February 2019 to December 2020. ^a^Chi-square test. ^b^Fisher test. ^c^Not considered for the estimation of the association.
(DOCX)

## References

[1] Hosek SG, Rudy B, Landovitz R, Kapogiannis B, Siberry G, Rutledge B, et al. An HIV Preexposure Prophylaxis Demonstration Project and Safety Study for Young MSM. J Acquir Immune Defic Syndr. 2017 Jan;74(1):21–9. doi: 10.1097/QAI.0000000000001179 27632233 PMC5140725

[2] Hosek SG, Landovitz RJ, Kapogiannis B, Siberry GK, Rudy B, Rutledge B, et al. Safety and feasibility of antiretroviral preexposure prophylaxis for adolescent men who have sex with men aged 15 to 17 years in the United States. JAMA Pediatr [Internet]. 2017;171(11):1063–71. Available from: https://jamanetwork.com/ doi: 10.1001/jamapediatrics.2017.2007 28873128 PMC5710370

[3] Schaefer R, Schmidt HMA, Ravasi G, Mozalevskis A, Rewari BB, Lule F, et al. Adoption of guidelines on and use of oral pre-exposure prophylaxis: a global summary and forecasting study. Lancet HIV [Internet]. 2021;8(8):e502–10. Available from: https://www.sciencedirect.com/science/article/pii/S2352301821001272 doi: 10.1016/S2352-3018(21)00127-2 34265283 PMC8332196

[4] Secretaria de Ciência, Tecnologia e Insumos Estratégicos, Ministério da Saúde. Portaria No21, de 25 de maio de 2017. Torna pública a decisão de incorporar o tenofovir associado a entricitabina (TDF/FTC 300/200mg) como profilaxia pré-exposição (PrEP) para [Internet]. Brasil; Available from: https://bvsms.saude.gov.br/bvs/saudelegis/sctie/2017/prt0021_29_05_2017.html

[5] Brasil. Protocolo clínico e diretrizes terapêuticas para Profilaxia Pré-exposição (PrEP) de risco à infecção pelo HIV. Ministério da Saúde Brasília; 2022.

[6] Grant RM, Anderson PL, McMahan V, Liu A, Amico KR, Mehrotra M, et al. Uptake of pre-exposure prophylaxis, sexual practices, and HIV incidence in men and transgender women who have sex with men: a cohort study. Lancet Infect Dis. 2014 Sep;14(9):820–9. doi: 10.1016/S1473-3099(14)70847-3 25065857 PMC6107918

[7] Anderson PL, Glidden D V, Liu A, Buchbinder S, Lama JR, Guanira JV, et al. Emtricitabine-tenofovir concentrations and pre-exposure prophylaxis efficacy in men who have sex with men. Sci Transl Med. 2012 Sep;4(151):151ra125. doi: 10.1126/scitranslmed.3004006 22972843 PMC3721979

[8] Haberer JE, Mugo N, Bukusi EA, Ngure K, Kiptinness C, Oware K, et al. Understanding Pre-Exposure Prophylaxis Adherence in Young Women in Kenya. J Acquir Immune Defic Syndr. 2022;89(3):251–60. doi: 10.1097/QAI.0000000000002876 35147580 PMC8826617

[9] Wheeler DP, Fields SD, Beauchamp G, Chen YQ, Emel LM, Hightow‐Weidman L, et al. Pre‐exposure prophylaxis initiation and adherence among Black men who have sex with men (MSM) in three US cities: results from the HPTN 073 study. J Int AIDS Soc. 2019;22(2):e25223. doi: 10.1002/jia2.25223 30768776 PMC6376611

[10] Zhang J, Li C, Xu J, Hu Z, Rutstein SE, Tucker JD, et al. Discontinuation, suboptimal adherence, and reinitiation of oral HIV pre-exposure prophylaxis: a global systematic review and meta-analysis. Lancet HIV [Internet]. 2022;9(4):e254–68. Available from: doi: 10.1016/S2352-3018(22)00030-3 35364026 PMC9124596

[11] Hannaford A, Arens Y, Koenig H. Real-Time Monitoring and Point-of-Care Testing: A Review of the Current Landscape of PrEP Adherence Monitoring. Patient Prefer Adherence. 2021;15:259–69. doi: 10.2147/PPA.S248696 33574659 PMC7873020

[12] Williams AB, Amico KR, Bova C, Womack JA. A proposal for quality standards for measuring medication adherence in research. AIDS Behav. 2013 Jan;17(1):284–97. doi: 10.1007/s10461-012-0172-7 22407465 PMC3434290

[13] Osterberg L, Blaschke T. Adherence to medication. N Engl J Med. 2005 Aug;353(5):487–97. doi: 10.1056/NEJMra050100 16079372

[14] Marins LMS, Torres TS, Leite I da C, Moreira RI, Luz PM, Hoagland B, et al. Performance of HIV pre-exposure prophylaxis indirect adherence measures among men who have sex with men and transgender women: Results from the PrEP BrasIL study. PLoS One. 2019;14(8):1–12. doi: 10.1371/journal.pone.0221281 31430318 PMC6701758

[15] Stirratt MJ, Dunbar-Jacob J, Crane HM, Simoni JM, Czajkowski S, Hilliard ME, et al. Self-report measures of medication adherence behavior: recommendations on optimal use. Transl Behav Med. 2015 Dec;5(4):470–82. doi: 10.1007/s13142-015-0315-2 26622919 PMC4656225

[16] Blumenthal J, Pasipanodya EC, Jain S, Sun S, Ellorin E, Morris S, et al. Comparing Self-Report Pre-Exposure Prophylaxis Adherence Questions to Pharmacologic Measures of Recent and Cumulative Pre-Exposure Prophylaxis Exposure. Front Pharmacol. 2019;10(July):1–8. doi: 10.3389/fphar.2019.00721 31333454 PMC6624646

[17] Leech AA, Biancarelli D, Aaron E, Miller ES, Coleman JS, Anderson PL, et al. HIV Pre-Exposure Prophylaxis for Conception Among HIV Serodiscordant Couples in the United States: A Cohort Study. 2020;34(7):295–302. doi: 10.1089/apc.2020.0005 32639209 PMC7370981

[18] Baker Z, Javanbakht M, Mierzwa S, Pavel C, Lally M, Zimet G, et al. Predictors of Over-Reporting HIV Pre-exposure Prophylaxis (PrEP) Adherence Among Young Men Who Have Sex With Men (YMSM) in Self-Reported Versus Biomarker Data. AIDS Behav. 2018 Apr;22(4):1174–83. doi: 10.1007/s10461-017-1958-4 29079950 PMC6038811

[19] Dourado I, Magno L, Greco DB, Zucchi EM, Ferraz D, Westin MR, et al. Interdisciplinarity in HIV prevention research: the experience of the PrEP1519 study protocol among adolescent MSM and TGW in Brazil. Cad Saude Publica [Internet]. 2023;39(suppl 1). Available from: http://www.scielo.br/scielo.php?script=sci_arttext&pid=S0102-311X2023001304001&tlng=en doi: 10.1590/0102-311XEN143221 36995865

[20] Anderson PL, Liu AY, Castillo-Mancilla JR, Gardner EM, Seifert SM, McHugh C, et al. Intracellular Tenofovir-Diphosphate and Emtricitabine-Triphosphate in Dried Blood Spots following Directly Observed Therapy. Antimicrob Agents Chemother. 2018 Jan;62(1). doi: 10.1128/AAC.01710-17 29038282 PMC5740314

[21] Yager J, Castillo-Mancilla J, Ibrahim ME, Brooks KM, McHugh C, Morrow M, et al. Intracellular Tenofovir-Diphosphate and Emtricitabine-Triphosphate in Dried Blood Spots Following Tenofovir Alafenamide: The TAF-DBS Study. J Acquir Immune Defic Syndr. 2020 Jul;84(3):323–30. doi: 10.1097/QAI.0000000000002354 32539288

[22] Hajian-Tilaki K. Sample size estimation in diagnostic test studies of biomedical informatics. J Biomed Inform. 2014 Apr;48:193–204. doi: 10.1016/j.jbi.2014.02.013 24582925

[23] Hajian-Tilaki K. The choice of methods in determining the optimal cut-off value for quantitative diagnostic test evaluation. Stat Methods Med Res. 2018 Aug 1;27(8):2374–83. doi: 10.1177/0962280216680383 28673124

[24] Obuchowski NA. Nonparametric analysis of clustered ROC curve data. Biometrics. 1997;567–78. 9192452

[25] Liu H, Wu T. Estimating the area under a receiver operating characteristic curve for repeated measures design. J Stat Softw. 2003;8:1–18.

[26] Amico KR, Marcus JL, McMahan V, Liu A, Koester KA, Goicochea P, et al. Study product adherence measurement in the iPrEx placebo-controlled trial: concordance with drug detection. J Acquir Immune Defic Syndr. 2014 Aug;66(5):530–7. doi: 10.1097/QAI.0000000000000216 24853306 PMC4110718

[27] Oliveira RL e S e, Silva LAV da, Duarte FM, Brasil SA, Castellanos MEP, Magno L, et al. Building bridges to care: the experience of peer navigation in enabling linkage to PrEP for adolescent men who have sex with men and transgender women. Cad Saude Publica [Internet]. 2023;39(suppl 1). Available from: http://www.scielo.br/scielo.php?script=sci_arttext&pid=S0102-311X2023001305006&tlng=en doi: 10.1590/0102-311XEN176821 36995868

[28] Musinguzi N, Muganzi CD, Boum Y 2nd, Ronald A, Marzinke MA, Hendrix CW, et al. Comparison of subjective and objective adherence measures for preexposure prophylaxis against HIV infection among serodiscordant couples in East Africa. AIDS. 2016 Apr;30(7):1121–9. doi: 10.1097/QAD.0000000000001024 26785125

[29] Abaasa A, Hendrix C, Gandhi M, Anderson P, Kamali A, Kibengo F, et al. Utility of Different Adherence Measures for PrEP: Patterns and Incremental Value. AIDS Behav [Internet]. 2018;22(4):1165–73. Available from: doi: 10.1007/s10461-017-1951-y 29090394 PMC5878836

