# Supplementary material for: Performance of indirect adherence measures for daily oral pre-exposure prophylaxis for HIV among adolescent men who have sex with men and transgender women in Brazil
Source: PLoS One. 2024 Dec 31;19(12):e0310861. doi: 10.1371/journal.pone.0310861 (PMC11687640; doi:10.1371/journal.pone.0310861)
Supplement: S1 Table — (DOCX) [file pone.0310861.s001.docx]

**S1 Table. Distribution of DBS Collection Weeks by Follow-Up Visit Number for Randomly Selected AMSM**

| **Follow-up visit number** | **Number of observations** | **Week of DBS collection** | | | |
| --- | --- | --- | --- | --- | --- |
|  |  | **Mean** | **Median** | **Min** | **Max** |
| 1 | 28 | 7.81 | 4.12 | 1.00 | 55.86 |
| 2 | 30 | 20.34 | 16.07 | 7.00 | 64.71 |
| 3 | 30 | 35.23 | 29.93 | 14.57 | 74.71 |
| 4 | 30 | 49.70 | 44.00 | 14.10 | 24.43 |
| 5 | 30 | 61.02 | 56.57 | 48.28 | 95.43 |
| 6 | 36 | 70.13 | 67.28 | 46.00 | 89.86 |
