# Supplementary material for: Performance of indirect adherence measures for daily oral pre-exposure prophylaxis for HIV among adolescent men who have sex with men and transgender women in Brazil
Source: PLoS One. 2024 Dec 31;19(12):e0310861. doi: 10.1371/journal.pone.0310861 (PMC11687640; doi:10.1371/journal.pone.0310861)
Supplement: S2 Table — PrEP1519 study, February 2019 to December 2020. aInterval notation is used to describe categories: parentheses indicate that the number is excluded from the interval, while square brackets indicate that the number is included in the interval. (DOCX) [file pone.0310861.s002.docx]

**S2 Table.** **Distribution of DBS Samples by collection week. PrEP1519 study, February 2019 to December 2020.**

| **Week of DBS collection ^a^** | **n (%)** n=302 |
| --- | --- |
| [1, 4] | 26 (8.61) |
| (4, 12] | 38 (12.58) |
| (12, 24] | 57 (18.87) |
| (24 to 36] | 37 (12.25) |
| (36 to 48] | 40 (13.25) |
| (48 to 60] | 34 (11.26) |
| (60 to 72] | 47 (15.56) |
| (72, 96] | 23 (7.62) |
| ^a^ Interval notation is used to describe categories: parentheses indicate that the number is excluded from the interval, while square brackets indicate that the number is included in the interval. | |
