# Supplementary material for: Performance of indirect adherence measures for daily oral pre-exposure prophylaxis for HIV among adolescent men who have sex with men and transgender women in Brazil
Source: PLoS One. 2024 Dec 31;19(12):e0310861. doi: 10.1371/journal.pone.0310861 (PMC11687640; doi:10.1371/journal.pone.0310861)
Supplement: S3 Table — PrEP1519 study, February 2019 to December 2020. aChi-square test. bFisher test. cNot considered for the estimation of the association. (DOCX) [file pone.0310861.s003.docx]

**S3 Table. Comparison of participants’ baseline characteristics included and not included in the accuracy analysis, by subpopulation. PrEP1519 study, February 2019 to December 2020.**

| **Characteristics** | **Men who have sex with men** | | | **Transgender women** | | |
| --- | --- | --- | --- | --- | --- | --- |
|  | **Included** | **Not included** | **p value** | **Included** | **Not included** | **p value** |
| Age |  |  | 0.650^a^ |  |  | 0.756^a^ |
| 15 - 17 years | 26 (21.49) | 95 (78.51) |  | 11 (68.75) | 5 (31.25) |  |
| 18 - 19 years | 122 (23.42) | 399 (76.58) |  | 29 (64.44) | 16 (35.56) |  |
| Skin-color |  |  | 0.904^a^ |  |  | 0.581^a^ |
| White | 43 (23.37) | 141 (76.63) |  | 8 (72.73) | 3 (27.27) |  |
| Non-White | 105 (22.93) | 353 (77.07) |  | 32 (64.00) | 18 (36.00) |  |
| Study site |  |  | 0.019^a^ |  |  | 0.004^b^ |
| Belo Horizonte | 40 (28.99) | 98 (71.01) |  | 13 (100.00) | 0 (0.00) |  |
| Salvador | 51 (26.42) | 142 (73.58) |  | 13 (65.00) | 7 (35.00) |  |
| São Paulo | 57 (18.33) | 254 (81.67) |  | 14 (50.00) | 14 (50.00) |  |
| Schooling |  |  | 0.428^a^ |  |  | 0.308^b^ |
| Higher education | 39 (21.31) | 144 (78.69) |  | 5 (100.00) | 0 (0.00) |  |
| High school or less | 107 (24.26) | 334 (75.74) |  | 35 (67.31) | 17 (32.69) |  |
| Not available^c^ | 2 (11.11) | 16 (88.89) |  | 0 (0.00) | 4 (100.00) |  |
| Condomless anal sex |  |  | 0.649^a^ |  |  | 0.217^b^ |
| No | 51 (22.47) | 176 (77.53) |  | 12 (60.00) | 8 (40.00) |  |
| Yes | 97 (24.07) | 306 (75.93) |  | 28 (75.68) | 9 (24.32) |  |
| Not available^c^ | 0 (0.00) | 12 (100.00) |  | 0 (0.00) | 4 (100.00) |  |
| Partner living with HIV |  |  | 0.144^a^ |  |  | 0.271^b^ |
| No/Don’t know | 113 (24.30) | 352 (75.70) |  | 35 (74.47) | 12 (25.53) |  |
| Yes | 13 (35.14) | 24 (64.86) |  | 0 (0.00) | 1 (100.00) |  |
| Not available^c^ | 22 (15.71) | 118 (84.29) |  | 5 (38.46) | 8 (61.54) |  |
| ^a^Chi-square test. ^b^Fisher test. ^c^Not considered for the estimation of the association | | | | | | |
